# Immunological and prognostic analysis of PSENEN in low-grade gliomas: An immune infiltration-related prognostic biomarker

**DOI:** 10.3389/fnmol.2022.933855

**Published:** 2022-07-28

**Authors:** Kaijie Chen, Beibei Liang, Wenhao Ma, Guoqing Wan, Bing Chen, Changlian Lu, Yuzhou Luo, Xuefeng Gu

**Affiliations:** ^1^Shanghai Key Laboratory of Molecular Imaging, Zhoupu Hospital, Shanghai University of Medicine and Health Sciences, Shanghai, China; ^2^School of Health Science and Engineering, The University of Shanghai for Science and Technology, Shanghai, China; ^3^School of Pharmacy, Shanghai University of Medicine and Health Sciences, Shanghai, China; ^4^Department of Neurosurgery, Affiliated Hospital of Guangdong Medical University, Zhanjiang, China; ^5^Business School, Guilin University of Technology, Guilin, China

**Keywords:** PEN2 (PSENEN), metformin, low-grade glioma, prognosis, immune infiltration, macrophage M2

## Abstract

Metformin is widely used in the treatment of type 2 diabetes (T2D) and plays a role in antitumor and antiobesity processes. A recent study identified its direct molecular target, PEN2 (PSENEN). PSENEN is the minimal subunit of the multiprotein complex γ-secretase, which promotes the differentiation of oligodendrocyte progenitors into astrocytes in the central nervous system. This study was mainly based on gene expression data and clinical data from the TCGA and CGGA databases. Analysis of differential expression of PSENEN between tissues from 31 cancers and paracancerous tissues revealed that it had high expression levels in most cancers except 2 cancers. Using univariate Cox regression analysis and Kaplan-Meier survival analysis, a high expression level of PSENEN was shown to be a risk factor in low-grade gliomas (LGG). Gene ontology (GO) and kyoto encyclopedia of genes and genomes (KEGG) analyses indicated that PSENEN is widely involved in immune-related signaling pathways in LGG. PSENEN expression level was significantly associated with TMB, MSI, tumor stemness index, and the expression levels of immunomodulatory genes in LGG. Finally, immune infiltration analysis revealed that PSENEN level was associated with the presence of various immune infiltrating cells, among which PSENEN was strongly associated with the presence of M2 macrophages and played a synergistic pro-cancer role. In conclusion, PSENEN may partially influence prognosis by modulating immune infiltration in patients with LGG, and PSENEN may be a candidate prognostic biomarker for determining prognosis associated with immune infiltration in LGG.

## Introduction

Metformin, a derivative of biguanide, has been commonly used for the treatment of T2D over the past century (Bailey, 2017). Metformin inhibits hepatic gluconeogenesis in both AMPK-dependent and AMPK-independent pathways, thus, exerting its anti-hyperglycemic effect (Fullerton et al., 2013). In recent years, studies have also found that metformin has many therapeutic benefits in anti-aging and anti-cancer (Podhorecka et al., 2017). For instance, metformin may slow the aging process by reducing DNA damage (Algire et al., 2012) and decreasing cellular inflammation (Valencia et al., 2017). Glioma is the most common primary intracranial tumor (Ma et al., 2021). Metformin can be used as a cancer stem cell initiation-targeting agent against glioblastoma cancer stem cells and brain tumor cells (Sato et al., 2012; Al Hassan et al., 2018). Specifically, metformin induces the differentiation of stem cell-like glioma-initiating cells through the activation of FOXO3 by AMP-activated protein kinase (AMPK). The synergistic effect of metformin and temozolomide has been reported to inhibit the proliferation of glioma cells, including glioblastoma multiforme (Yang et al., 2016). Although metformin has a long history of use and is effective in many diseases, its direct molecular target, PEN2 (PSENEN), has not been discovered until recently (Ma et al., 2022).

The PSENEN is an essential subunit of the multiprotein complex γ-secretase and, together with APH1, presenilin, and nicastrin, regulate amyloid beta-peptide (Abeta) levels and γ-secretase activity (Kimberly et al., 2003; Luo et al., 2003; Marlow et al., 2003). Among them, γ-secretase catalyzes the intramembrane cleavage of β-amyloid precursor protein (APP) to release Abeta, the major component of senile plaques associated with Alzheimer’s disease (AD) (Edbauer et al., 2003). In connection with this, studies suggest that PSENEN mutations may be associated with Alzheimer’s disease (Andreoli et al., 2011). Furthermore, several reports suggest that PSENEN mutations are involved in the occurrence and development of hidradenitis suppurativa (HS) and Dowling-Degos disease (DDD). Specifically, Xiao et al. (2020) found that PSENEN mutations in patients with HS predispose them to DDD by disrupting Notch signaling. Li et al. (2018) observed that the location of PSENEN mutations in patients with HS all occurred in transmembrane regions that are expected to disrupt membrane function. Ralser et al. (2017) showed that PSENEN mutations underlie a DDD subtype with increased susceptibility to HS. Pavlovsky et al. (2018) suggested the coexistence of DDD and HS is caused by PSENEN mutations. On the other hand, PSENEN not only plays an important role in adipocyte differentiation (Lee et al., 2009) but also promotes the differentiation of oligodendrocyte progenitor cells into astrocytes in the central nervous system (Hou et al., 2021). Concretely, the deletion of PSENEN inhibits HES1 and activates STAT3 to trigger GFAP activation, thereby, promoting astrocyte differentiation. However, few studies have focused on the prognostic and immunological value of PSENEN in human cancers.

In this study, we first explored the differences in the expression of PSENEN between tissues from 31 cancers and corresponding normal tissues. To reveal the potential prognostic value between PSENEN expression and patients with low-grade glioma, a survival analysis was performed based on TCGA and CGGA databases. Interestingly, gene ontology (GO) and kyoto encyclopedia of genes and genomes (KEGG) analyses found that PSENEN was extensively involved in immunoregulatory signaling pathways in LGG. Subsequently, we investigated the relationship of PSENEN expression with levels of immune-related factors and the presence of immune-infiltrating cells in LGG in a multifaceted manner. Altogether, this study focuses on the prognostic value and immunological insights into the functions played by PSENEN in LGG, which may support further molecular mechanistic studies.

## Materials and methods

### Data acquisition

The RNA expression data and clinical data from the ‘‘GDC TCGA Lower Grade Glioma (LGG)’’ cohort were downloaded on the UCSC Xena database^1^ (Goldman et al., 2020). To obtain more gene expression data from paracancerous tissue samples, we got the PSENEN gene expression data, which combined TCGA and GTEx databases of 31 cancers on the UCSCXenaShiny portal^2^ (Wang S. et al., 2021). The Chinese Glioma Genome Atlas (CGGA) is a data portal storing multi-omics data and detailed clinical information on nearly 2,000 primary and recurrent glioma samples from a Chinese cohort (Zhao et al., 2021). The expression data and clinical data of mRNAseq_693 and mRNAseq_325 datasets were downloaded through the CGGA “Download” interface and integrated via R 4.1.0. The gene expression profiles of GSE4290 datasets (Sun et al., 2006) were downloaded from the GEO database.^3^ Gene Expression Profiling Interactive Analysis (GEPIA)^4^ webserver is a user-friendly web portal for gene expression analysis based on TCGA and GTEx databases (Li et al., 2021). The “Expression DIY” module of GEPIA was used to study the differential expression of PSENEN in LGG tissues and normal tissues.

### Immunohistochemical staining and subcellular localization

The Human Protein Atlas^5^ focuses on antibody-based proteomics and integrated omics, collecting tens of millions of immunohistochemical (IHC) images of human proteins in multiple organs and different cancers (Thul and Lindskog, 2017). The COMPARTMENTS database integrates and automatically synchronizes multiple databases, prediction tools, and literature to provide subcellular localization of gene proteins (Binder et al., 2014). Antibody-based immunohistochemical protein profiling and subcellular localization details of PSENEN protein in LGG were obtained using the HPA database and COMPARTMENTS database, respectively.

### Survival prognosis analysis

Univariate Cox regression models were used to investigate whether PSENEN expression was an independent prognostic indicator for patients with LGG and its subtypes. Kaplan-Meier curves were used to assess the prognostic significance of PSENEN expression. Clinical survival data included overall survival (OS), disease-specific survival (DSS), disease-free interval (DFI), and progression-free interval (PFI).

### Gene ontology functional analysis and kyoto encyclopedia of genes and genomes pathway enrichment analysis

GO and KEGG analyses were applied to explore the biological functions of PSENEN in LGG. Based on the median PSENEN gene expression level, the TCGA-LGG cohort was divided into PSENEN high and low expression groups to perform differential gene expression analysis. Then, GO and KEGG analyses were performed on the differentially expressed gene (DEG) sets obtained using the R package ClusterProfiler (Wu et al., 2021).

### Correlation analysis of PSENEN expression with tumor mutational burden, microsatellite instability, tumor stemness index, and immunomodulatory gene

Tumor mutational burden (TMB) refers to the number of somatic mutations in the tumor genome after the removal of germline mutations (Merino et al., 2020). Microsatellite instability (MSI) describes the emergence of a new microsatellite allele at a microsatellite locus in a tumor due to the insertion or deletion of a repetitive unit (Dudley et al., 2016). Tumor stemness indices are indicators for assessing the degree of oncogenic dedifferentiation. Among them, mRNAsi is a gene expression-based stemness index, while mDNAsi is a DNA methylation-based stemness index (Malta et al., 2018). Immunomodulatory genes were obtained from the TISIDB database^6^ (Ru et al., 2019). Correlations between PSENEN expression and TMB, MSI, mRNAsi, mDNAsi, and immunomodulatory genes were analyzed using Pearman’s method.

### Immune infiltration analysis

TIMER^7^ is a user-friendly web interface for comprehensive analysis of gene and tumor-infiltrating immune cells and visualization of their associations (Li et al., 2017). The relationship between PSENEN expression and the levels of infiltration of six immune cells (B cells, CD4 + T cells, CD8 + T cells, neutrophils, macrophages, and dendritic cells) in LGG was examined using the TIMER database. ESTIMATE is an algorithm that infers the fraction of stromal and immune cells in tumor samples using gene expression profiles (Yoshihara et al., 2013). CIBERSORT-ABS is a method to estimate the abundance of immune cells by deconvoluting the expression matrix of immune cell subtypes using the principle of linear support vector regression (Newman et al., 2015). Correlations analysis between PSENEN expression and ESTIMATE scores or 22 types of infiltrating immune cells were performed using Spearman’s method.

## Results

### PSENEN expression is increased in low-grade gliomas

**FIGURE 1:**
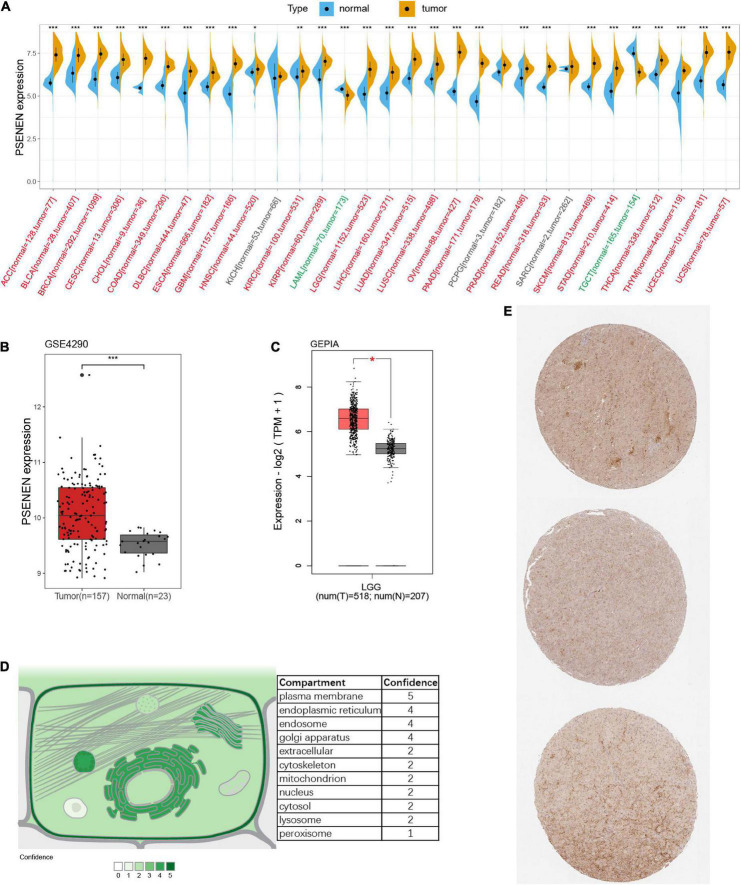
Gene expression and protein information of PSENEN. **(A)** Differential expression of PSENEN in normal and tumor samples from patients with 31 types of cancers. Read labels on the *x*-axis represent an elevated expression of PSENEN in tumors compared to normal tissues, while green labels represent diminished expression, and black labels indicate no significant difference. PSENEN expression in LGG was examined by using the GSE4290 dataset **(B)** and GEPIA database **(C)**. **(D)** Subcellular locations of PSENEN from the COMPARTMENTS database. **(E)** Representative PSENEN immunohistochemical staining in LGG tissues. “*”, “**” and “***” indicate *p* < 0.05, *p* < 0.01, and *p* < 0.001, respectively.

As illustrated in Figure 1A, significantly higher PSENEN expression levels were detected in most human cancers than in adjacent normal tissues, such as ACC, BLCA, BRCA, CESC, CHOL, COAD, DLBC, ESCA, GBM, HNSC, KIRC, KIRP, LGG, LIHC, LUAD, LUSC, OV, PAAD, PRAD, READ, SKCM, STAD, THCA, THYM, UCEC, and UCS. In contrast, significantly lower PSENEN expression levels were observed in a few human cancers (LAML and TGCT). Conformably, we also found that PSENEN was expressed at higher levels in LGG tissue than in normal tissue in the GSE4290 cohort and GEPIA databases (Figures 1B,C). Subcellular localization is an indispensable technical means to study the function of genes, which can identify the specific location of proteins in cells. PSENEN proteins are mainly localized in the plasma membrane, endoplasmic reticulum, endosome, and Golgi apparatus (Figure 1D). Finally, PSENEN expression was detected in clinical low-grade glioma specimens (Figure 1E).

### Increased PSENEN expression corresponds with a poor prognosis in low-grade gliomas

**FIGURE 2:**
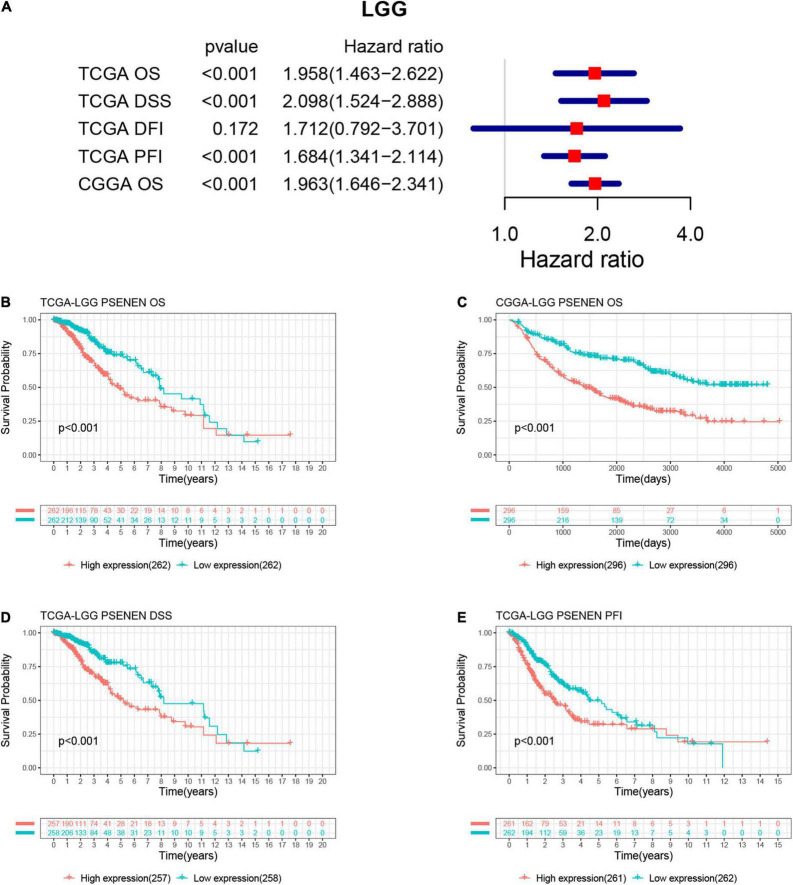
Associations between PSENEN expression levels and the survival prognosis of patients with LGG. **(A)** Forest plot showing the hazard ratios of PSENEN in LGG. Kaplan—Meier survival curves of OS **(B,C)**, DSS **(D),** and PFI **(E)** for patients stratified according to PSENEN expression profiles in LGG.

We estimated the association between PSENEN expression levels and the prognosis of patients with low-grade glioma in the TCGA and CGGA databases. As indicated in Figure 2, a univariate Cox regression analysis of OS, DSS, and PFI suggested that increased PSENEN expression was a risk factor in LGG. In addition, the Kaplan-Meier survival curves indicated that increased PSENEN expression was remarkably associated with shorter OS, DSS, and PFI in patients with LGG.

### PSENEN expression level is significantly correlated with the clinical and molecular features of low-grade gliomas

**FIGURE 3:**
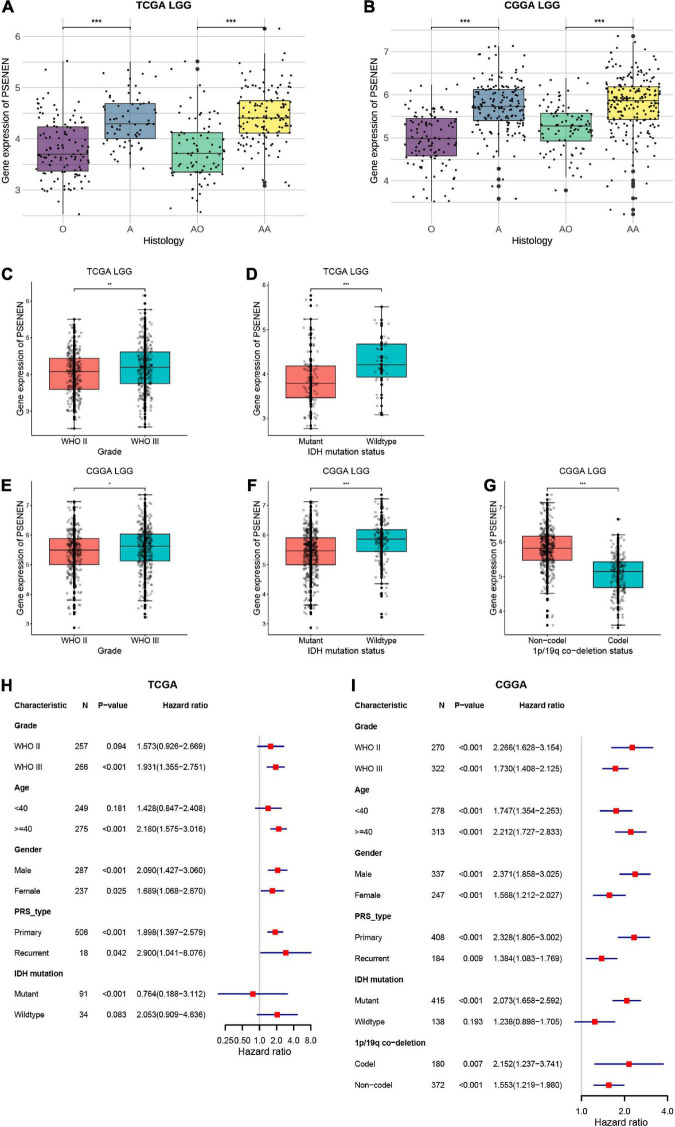
Differential expression analysis and prognosis analysis of PSENEN in clinical subtypes of LGG. **(A,B)** The correlation of PSENEN expression level with histology in LGG of TCGA and CGGA databases. **(C–G)** The relationship between the PSENEN transcription level and WHO grade, IDH mutation, and 1p/19q codeletion in the CGGA and TCGA databases. **(H,I)** Forest plots show the correlation between PSENEN expression and clinical parameters in LGG from the TCGA and CGGA databases. “*”, “**” and “***” indicate *p* < 0.05, *p* < 0.01, and *p* < 0.001, respectively.

In general, low-grade gliomas have been morphologically classified into oligodendroglioma (O) and astrocytoma (A) (van den Bent et al., 2017). Among these subgroups, there are anaplastic oligodendrogliomas (AO) and anaplastic astrocytomas (AA) according to the malignancy grade (WHO III) (Wesseling and Capper, 2018). To explore the role of PSENEN in the characteristics of human glioma, we analyzed its expression levels in LGG samples from the TCGA and CGGA databases. PSENEN expression levels were significantly increased in astrocytomas compared to oligodendrogliomas (Figures 3A,B) and further increased along with grade II to III progression (Figures 3C,E). IDH mutation and 1p/19q codeletion are unique and are clinically significant molecular features in low-grade gliomas (Dono et al., 2021; McAleenan et al., 2022). We, therefore, investigated the relationship between PSENEN expression level and the status of IDH mutation and 1p/19q codeletion. As indicated in Figures 3D,F, significantly lower PSENEN expression levels were detected in IDH-mutant than in patients with IDH wildtype. Additionally, low PSENEN expression levels were observed in the 1p/19q codeletion, and high expression levels were observed in the patients with 1p/19q wildtype (Figure 3G). To better understand the prognostic value and potential mechanism of PSENEN expression in low-grade glioma, we explored the association between PSENEN transcription levels and clinical characteristics using univariate Cox regression analysis. Consistently, higher PSENEN expression levels were significantly associated with poorer OS in patients with different subtypes of low-grade gliomas, including grade II and grade III patients, male and female patients, and primary and recurrent patients (Figures 3H,I).

### PSENEN is involved in the immune-inflammatory response

**FIGURE 4:**
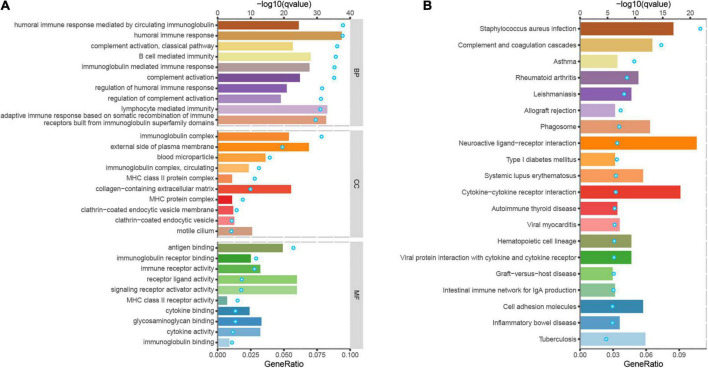
Gene ontology (GO) and kyoto encyclopedia of genes and genomes (KEGG) analyses of DEGs between PSENEN high expression and low expression groups in LGG. **(A)** Top 10 BP, CC, and MF enrichment terms of DEGs. **(B)** Top 20 KEGG enrichment pathways of DEGs. The blue dots represent the *q*-values of the upper axis enrichment pathways, and the horizontal bars correspond to the gene ratio in the lower axis enrichment terms.

To further investigate the molecular mechanisms of PSENEN regulation in LGG, we performed GO and KEGG analyses (Figure 4). Notably, in terms of biological processes (BP), PSENEN functions were enriched in immune response-related processes, such as complement activation, humoral immune response, B-cell-mediated immunity, and lymphocyte-mediated immunity. The cellular component (CC) pathways were mainly involved in the immunoglobulin complex and MHC protein complex. The molecular function (MF) mainly involved antigen binding, immune receptor activity, and receptor-ligand activity. Furthermore, the top 20 significant terms of the KEGG analysis included staphylococcus aureus infection, complement and coagulation cascades, hematopoietic cell lineage, the intestinal immune network for IgA production, and so on. These results suggest that PSENEN plays an important role in the inflammatory response and tumor immune microenvironment.

### PSENEN expression is related to multiple immune-related factors

**FIGURE 5:**
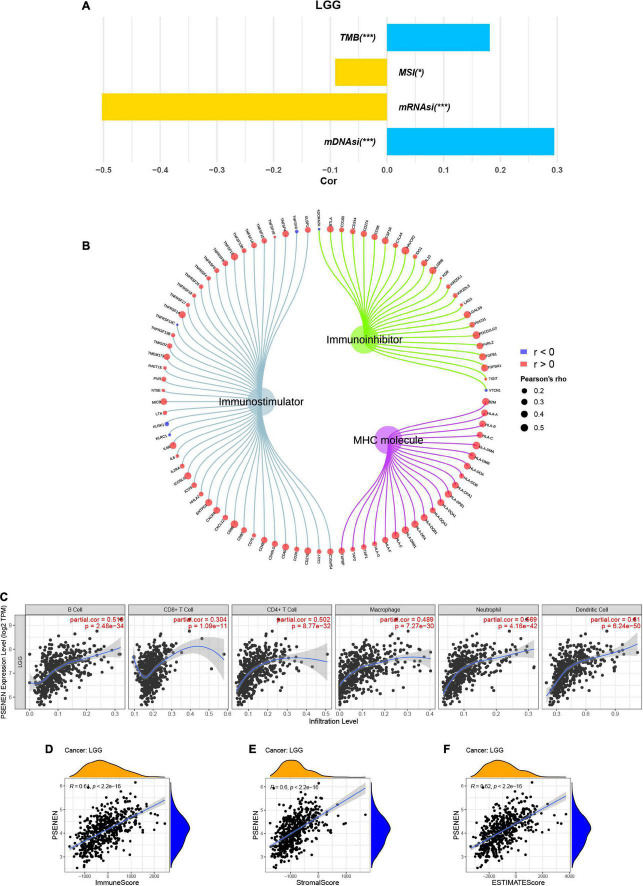
Correlations between PSENEN expression and multiple immune-related factors in LGG. **(A)** The relationship between PSENEN expression and TMB, MSI, mRNAsi, and mDNAsi. **(B)** Correlation of PSENEN expression level with levels of immunostimulator, immuno-inhibitory, and MHC molecule-related genes. **(C)** Correlation of PSENEN expression with immune cell infiltration levels. Correlation of PSENEN expression with **(D)** ImmuneScore, **(E)** StromalScore and **(F)** ESTIMATEScore in LGG. ‘*’ and ‘***’ indicate *p* < 0.05 and *p* < 0.001, respectively.

TMB, MSI, and tumor stemness indices are associated with the immune checkpoint inhibitor (ICI) response and might predict the efficacy of tumor immunotherapy (Dudley et al., 2016; Malta et al., 2018; Ritterhouse, 2019). Thus, we investigated the relationship between PSENEN expression and TMB, MSI, and tumor stemness indices. PSENEN expression in LGG was negatively correlated with the MSI and mRNAsi, while it was positively correlated with the TMB and mDNAsi (Figure 5A). In addition, the potential association of PSENEN expression levels with levels of immunomodulators (immuno-inhibitors, immunostimulators, and MHC molecules) was investigated. As shown in Figure 5B, PSENEN expression level was positively correlated with the level of 41 immunostimulators and negatively correlated with the level of 4 immunostimulators. The correlation analyses of immuno-inhibitors demonstrated that PSENEN expression was positively associated with most of them. Interestingly, PSENEN expression displayed a strong relationship with all types of MHC molecule-related genes. According to the results of the TIMER database, PSENEN expression was significantly and positively correlated with six tumor-infiltrating immune cells (B cells, CD4 + T cells, CD8 + T cells, macrophages, neutrophils, and dendritic cells) in LGG (Figure 5C). We also investigated the association between PSENEN expression and ESTIMATE scores, and the results indicated that their correlation coefficients were all greater than 0.6 (Figures 5D–F). Taken together, the analysis results strongly suggest that PSENEN may play an important role in the tumor immune microenvironment and may be involved in immune regulation in LGG.

### PSENEN is associated with M2 macrophages in low-grade gliomas

**FIGURE 6:**
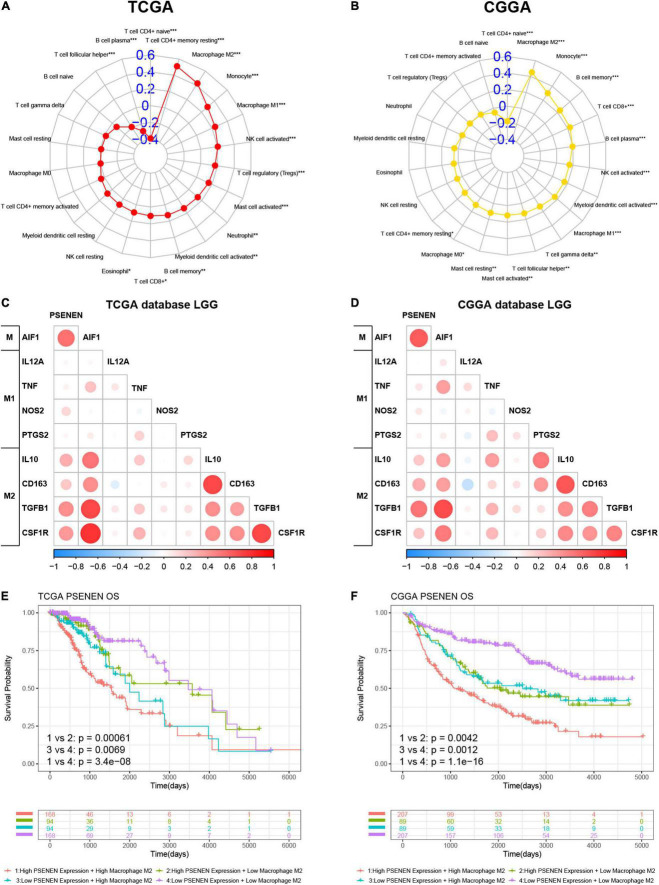
Association analysis of PSENEN expression level with levels of M2 macrophages in LGG from the TCGA and CGGA databases. **(A,B)** The correlation between PSENEN and 22 types of infiltrating immune cells. **(C,D)** Nine classical phenotype markers of M, M1, and M2 macrophages were analyzed. Color depth and circle square represent the degrees of correlation. **(E,F)** Combination survival analysis based on PSENEN expression and macrophages M2. “*”, “**”, and “***” indicate *p* < 0.05, *p* < 0.01, and *p* < 0.001, respectively.

CIBERSORT-ABS was used to explore the association between PSENEN gene expression and the infiltration of various subtypes of immune cells in LGG from the TCGA and CGGA databases. The radar charts demonstrate different associations between PSENEN expression and levels of various immune-infiltrating cells (Figures 6A,B). For example, PSENENE expression was significantly and positively correlated with the presence of M1 macrophages, M2 macrophages, and monocytes, but significantly and negatively related with the presence of naive CD4 + T cell in LGG from the TCGA and CGGA databases. Among them, the PSENEN expression level showed the strongest correlation with the presence of M2 macrophages (*R* > 0.4). The analysis of PSENEN expression level correlation with the levels of classical phenotypic markers of macrophage M (AIF1), M1 (IL12A, TNF, NOS2, and PTGS2), and M2 (IL10, CCL163, TGFB1, and CSF1R) (Ma et al., 2021) revealed a greater positive correlation with M and M2 markers than with M1 markers (Figures 6C,D). A combination survival analysis also showed that patients with high PSENEN expression and high levels of macrophage M2 infiltration had the worst overall survival (OS), while patients with low PSENEN expression and low levels of macrophage M2 infiltration had the best survival outcomes (Figures 6E,F). These findings suggest that PSENEN plays an important role in M2 macrophage infiltration and LGG progression.

## Discussion

By screening and repeatedly knocking down more than 2,000 targets, a recent study by Ma et al. (2022) finally identified the direct target of metformin, PEN2 (PSENEN), one of the subunits of the γ-secretase complex (Marlow et al., 2003). PSENEN is required for the activation of Notch signaling pathways (Vural et al., 2021). Also, multiple disease phenotypes are associated with PSENEN mutations, including Alzheimer’s disease (Andreoli et al., 2011), acne inversa (Li et al., 2018), and Dowling-Degos disease (Ralser et al., 2017). In this study, we systematically analyzed the prognostic and immunological value of PSENEN in LGG. First, the results of multiple data cohorts suggest that PSENEN is expressed at higher levels in LGG tissue than in normal tissue. Both univariate Cox regression analysis and Kaplan-Meier survival curves showed that increased PSENEN expression level was associated with poor prognosis.

IDH mutations and 1p/19q codeletions were identified as molecular diagnostic markers for glioma in the new 2016 WHO brain tumor classification (van den Bent et al., 2017). Patients with LGG with IDH mutation or 1p/19q codeletion have a more favorable prognosis (Dono et al., 2021). The clinical analysis showed that PSENEN expression levels were higher in astrocytomas, high-grade tumors, IDH wildtype, and 1p/19q wildtype tumors than in their corresponding phenotype. These clinical features associated with high PSENEN expression levels are all correlated with poor clinical outcomes (McAleenan et al., 2022). Therefore, it was concluded that elevated expression of PSENEN was strongly associated with poor prognosis. It could be used as an independent risk prognostic indicator for LGG.

The most used predictive biomarkers for ICI are PD-L1, MSI, and TMB (Wang Y. et al., 2021; Lai et al., 2022). In addition, a high tumor stemness index is associated with reduced PD-L1 expression in most cancers (Malta et al., 2018). In this study, both immunotherapy biomarkers (TMB and MSI) and tumor stemness index were shown to be significantly associated with PSENEN expression in LGG. Moreover, the expression levels of PSENEN exhibited a significantly positive correlation with most of the expression levels of immunostimulatory genes and MHC molecules. Furthermore, the GO and KEGG analyses showed that PSENEN was involved in immune response-related pathways, such as humoral immune response, MHC protein complex, immune receptor activity, and complement and coagulation cascades pathway. The MHC protein complex is engaged in intracellular antigen processing and presentation and is closely related to the immune response and immune regulation (Roche and Furuta, 2015). Additionally, the complement system is a mediator of the protein hydrolysis cascade reaction in plasma and innate immunity, which is a non-specific defense mechanism against pathogens (Bajic et al., 2015). These results suggest a strong association of PSENEN with immune response in LGG.

Tumor-infiltrating immune cells play a central role in tumor control and response to therapy (Chen and Mellman, 2017). Among them, naive CD4 + T cells play critical roles in maintaining immunocompetence throughout life (Silva and Sousa, 2016). After interaction with the MHC II protein complex, naive CD4 + T cells could be activated and differentiated into specific subtypes (Luckheeram et al., 2012). Our analysis indicated a negative correlation between PSENEN expression level and the presence of naive CD4 + T cells in LGG. In connection with the results of GO analysis, PSENEN may affect naive CD4 + T cell activation and differentiation by regulating the MHC class II protein complex. On the other side, macrophages differentiate into M1 and M2 types under the influence of the tumor’s microenvironment cytokines (Cassetta and Pollard, 2020). Generally, M1 macrophages play an antitumor role, while M2 macrophages promote the occurrence and development of tumors. Utilizing immunohistochemistry, De et al. (2016) elucidated that glioma-associated macrophages can exhibit different polarization states of M1 and M2 in gliomas. In the meantime, M2 macrophages predominate the immune microenvironment in gliomas and lead to the suppression of local and systemic immunity (Vidyarthi et al., 2019). Our in-depth immune infiltration analysis revealed a strong correlation between PSENEN expression level and the presence of macrophages M2, as well as the synergistic pro-cancer effect between them. This finding implies that PSENEN may be involved in the polarization of macrophages toward the M2 phenotype.

Overall, this research is the first to investigate the prognostic and immunological value of PSENEN in LGG, contributing to the understanding of its function in tumor development and its role in immunology. Elevated levels of PSENEN transcripts are a risk factor for low-grade gliomas. Thus, PSENEN inhibitors may be potential alternative therapeutic approaches. In addition, PSENEN is directly or indirectly involved in immune regulation in LGG, especially in the differentiation of tumor-associated macrophages toward M2-like macrophages. However, more experimental studies are required to explore the specific mechanisms of PSENEN action in LGG.

## Data availability statement

The datasets presented in this study can be found in online repositories. The names of the repository/repositories and accession number(s) can be found in the article.

## Author contributions

XG, YL, and CL conceived and designed the study. KC and BL were responsible for the collection and assembly of data, data analysis, and interpretation. KC and WM were involved in the writing of the manuscript. GW and BC provided help in revising the manuscript. All authors read and approved the final manuscript.

## Footnotes

1
https://xena.ucsc.edu/

2
https://shiny.hiplot.com.cn/ucsc-xena-shiny/

3
https://www.ncbi.nlm.nih.gov/geo/

4
http://gepia2.cancer-pku.cn/#index

5
https://www.proteinatlas.org/

6
http://cis.hku.hk/TISIDB/index.php

7
https://cistrome.shinyapps.io/timer/
